# The fecal iron pump: Global impact of animals on the iron stoichiometry of marine sinking particles

**DOI:** 10.1002/lno.11597

**Published:** 2020-10-06

**Authors:** Priscilla K. Le Mézo, Eric D. Galbraith

**Affiliations:** ^1^ Institut de Ciència i Tecnologia Ambientals (ICTA) Universitat Autonoma de Barcelona (UAB) Barcelona Spain; ^2^ Laboratoire de Météorologie Dynamique (LMD) / Institut Pierre Simon Laplace CNRS, Ecole Normale Supérieure, Université PSL, Ecole Polytechnique, Sorbonne Université Paris France; ^3^ Catalan Institution for Research and Advanced Studies (ICREA) Barcelona Spain; ^4^ Earth and Planetary Sciences McGill University Montreal Quebec Canada

## Abstract

The impact of marine animals on the iron (Fe) cycle has mostly been considered in terms of their role in supplying dissolved Fe to phytoplankton at the ocean surface. However, little attention has been paid to how the transformation of ingested food into fecal matter by animals alters the relative Fe‐richness of particles, which could have consequences for Fe cycling in the water column and for the food quality of suspended and sinking particles. Here, we compile observations to show that the Fe to carbon (C) ratio (Fe:C) of fecal pellets of various marine animals is consistently enriched compared to their food, often by more than an order of magnitude. We explain this consistent enrichment by the low assimilation rates that have been measured for Fe in animals, together with the respiratory conversion of dietary organic C to excreted dissolved inorganic C. Furthermore, we calculate that this enrichment should cause animal fecal matter to constitute a major fraction of the global sinking flux of biogenic Fe, a component of the marine iron cycle that has been previously unappreciated. We also estimate that this fecal iron pump provides an important source of Fe to marine animals via coprophagy, particularly in the mesopelagic, given that fecal matter Fe:C can be many‐fold higher than the Fe:C of local phytoplankton. Our results imply that the fecal iron pump is important both for global Fe cycling and for the iron nutrition of pelagic and mesopelagic communities.

Iron (Fe) is an essential element used to support many functional aspects of marine organisms, including photosynthesis, cellular respiration, and oxygen transport (Bury and Grosell 2003*a*; Marchetti and Maldonado 2016). Its scarcity in the ocean has been shown to limit phytoplankton growth, especially in the nitrate‐rich High Nutrient Low Chlorophyll regions in which the addition of Fe can boost primary production (Moore et al. 2013). Moreover, it has been suggested that low Fe concentrations can be limiting to marine animals, including zooplankton (Chen et al. 2011) and fish (Galbraith et al. 2019). Because of its importance as a trace nutrient, the efficiency with which Fe is recycled at the surface, instead of being lost to depth in sinking particles, plays an important role in determining the total primary productivity in Fe‐limited regions (Boyd and Ellwood 2010).

Heterotrophic consumers, including multicellular animals, have received attention as recyclers of Fe within the euphotic zone of Fe‐limited regions (e.g., Sarthou et al. 2008; Ratnarajah et al. 2018). For example, in an high nutrient, low chlorophyll region, Sarthou et al. (2008) showed that copepod grazing increases Fe recycling, which supports about half of the local phytoplankton demand for Fe, and Nuester et al. (2014) also highlighted that dissolved Fe released by grazing meso‐zooplankton is taken up by phytoplankton faster than inorganic Fe. The activity of consumers, especially zooplankton, therefore plays a major role in the recycling of Fe in the surface layer.

Yet, marine animals not only recycle Fe within the surface ocean, they also produce Fe‐bearing particulate fecal matter (i.e., fecal pellets), with overall consequences for the iron cycle that remain poorly defined. On one hand, the export of Fe by fecal pellets can contribute to Fe limitation in the surface ocean due to their large sinking speed (Schmidt et al. 1999) and sometimes refractory nature (Cabanes et al. 2017). But on the other hand, prior work has argued that fecal matter fertilizes the water with Fe when it is remineralized (Schmidt et al. 2016; Laglera et al. 2017), which might suggest an equivocal overall outcome on Fe recycling. Thus, although sinking fecal pellets can contribute to a significant part of the export of organic carbon (e.g., Turner 2015) and can have an Fe:C that is significantly different than other particles (e.g., Cabanes et al. 2017), few studies have considered the role of animals in the particulate Fe distribution in the water column (an exception being the observational study of Laglera et al. 2017). Furthermore, little consideration has been given to how animals might contribute to the ways in which the vertical distribution of particulate Fe differs from that of C, nitrogen, and phosphorus in the global ocean.

One distinctive feature of the vertical distribution of Fe is that its total concentration in particles tends to increase with depth. The Fe:C of sinking matter, determined from the sinking flux of particulate Fe vs. the sinking flux of particulate C, also generally increases with depth (e.g., Frew et al. 2006; Bressac et al. 2019). These observed enrichments of Fe in sinking particles are typically attributed to abiological process, primarily the scavenging of Fe onto sinking particles, sediment resuspension and/or water mass mixing. Fecal pellets are not widely considered to contribute to the vertical changes in particulate Fe:C due, at least in part, to the lack of a general expectation for how the Fe content of feces should differ from the Fe of animal food.

Fecal matter also serves as a food source for many organisms in the whole water column. (Bailey and Robertson 1982; Köster and Paffenhöfer 2017), but is proportionally more important in the mesopelagic and bathypelagic layers where coprophagy makes up a larger part of the overall diet (e.g., González and Smetacek 1994; Sampei et al. 2009). Thus, the nutritional status of mesopelagic organisms living at depth and consuming sinking fecal matter will be strongly influenced by the stoichiometry of this fecal matter. Interestingly, there is a relatively high abundance of mesopelagic fish observed in Fe‐limited high nutrient, low chlorophyll regions (e.g., Beamish et al. 1999; Moteki et al. 2011), where epipelagic fish are scarce (Galbraith et al. 2019), prompting the question of whether or not fecal pellets might alleviate Fe limitation among mesopelagic organisms compared to epipelagic ones.

In this article, we develop the hypothesis that Fe:C is consistently enriched in the fecal matter of animals, relative to the food they ingest, and that this plays an important role in setting the vertical distribution of biogenic Fe in the water column. We refer to this overall process as the “fecal iron pump.” We additionally propose that this fecal iron pump provides an important source of Fe nutrition to organisms, especially in the mesopelagic community. We provide an initial test of these hypotheses by compiling Fe:C measurements in feces as well as data on Fe:C in marine organisms and on Fe and C absorption and assimilation efficiencies. We also build a simple model to provide rough estimates of the global impact of fecal Fe‐enrichment on sinking particles in the ocean. Our results support a significant role for the fecal iron pump, both as an overlooked part of the global Fe cycle and as a source of this critical micronutrient to the mesopelagic community.

## Methods

### Conceptual basis for data analysis

**Fig 1:**
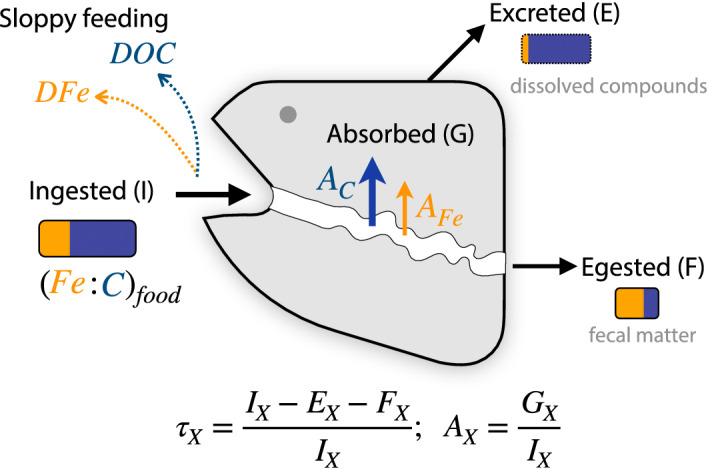
Simple representation of Fe:C stoichiometry transformations arising from the passage of food through a marine animal with a gut. The arrows represent the fluxes of elements. The colored boxes represent the Fe:C stoichiometry in the different components of the system. *τ*_*X*_ is the trophic efficiency for element X and *A*_*X*_ is the absorption efficiency for element X. Sloppy feeding releases dissolved organic Fe and C, DFe and DOC, respectively.

In order to analyze diverse data sources within a common framework, we first developed a framework within which to quantify the fates of ingested Fe and C in a generalized consumer, illustrated in Fig. 1. Part of the ingested food is absorbed through the gut epithelium, as determined by the absorption efficiency (also referred to elsewhere as the gut assimilation efficiency): we call *A*_*X*_ the absorption efficiency of an element X (Fig. 1). The food that is not absorbed is egested as fecal matter, (1 − *A*_*X*_)*I*_*X*_ where *I*_*X*_ is the quantity of X ingested. Part of the absorbed food is allocated to the construction of new biomass (including somatic growth and reproductive tissue), *α*_*X*_*A*_*X*_*I*_*X*_ where *α*_*X*_ is the somatic assimilation efficiency of an element X. The remainder is excreted, given by (1 − *α*_*X*_)*A*_*X*_*I*_*X*_. We call *τ*_*X*_ the trophic efficiency of an element X, so that the amount of element X incorporated in new matter is *τ*_*X*_*I*_*X*_ and *τ*_*X*_ = *A*_*X*_*α*_*X*_. Note that the trophic efficiency of C is sometimes called the gross growth efficiency. In this framework, the ingested fraction that we consider is the one that actually enters the digestive tract. We note that sloppy feeding, by which elements can be lost before entering the digestive tract as represented on Fig. 1 may also affect the Fe:C of the food and thus the measurements of efficiencies. For example, Møller et al. (2003) estimated for copepods that about 50% of particulate C is lost from food by sloppy feeding. We are not aware of any study quantifying the amount of Fe lost from the food via sloppy feeding and therefore assume that Fe and C are released in similar amounts during feeding, similar to Laglera et al. (2020).

Also, it has been shown that Fe can be taken up directly from water by fish via their gills, but this direct absorption is negligible at the low dissolved Fe concentrations found in seawater (Bury and Grosell 2003*b*). We therefore consider food as the only source of Fe to marine animals.

### Data on absorption and assimilation efficiencies of Fe and C

Table 1 shows data gathered from the literature on Fe and C absorption efficiencies for different organisms, and data on measured Fe assimilation or trophic efficiencies. For C assimilation efficiency, we refer to Sterner and Elser (2002) who give a C assimilation efficiency of 10–20% for marine animals.

**Table 1:** Absorption efficiencies of Fe and C, *A*_Fe_ and *A*_C_, and assimilation (or trophic) efficiency of Fe, *τ*_Fe_, for marine plankton and fish fed different diets. Maximum efficiencies equal 1. “Fe‐poor” and “Fe‐rich” indicate whether the food was cultivated under low or high Fe concentrations. Values in italic may have been biased by sloppy feeding.

Organism		Food	*A*_Fe_	*A*_C_	*τ*_Fe_	Reference
Calanoid copepod assemblage, mostly *Acartia* spp.		Fe‐poor *Thalassiosira weissflogii*	0.17	0.46	—	Schmidt et al. 1999
		Fe‐rich *Thalassiosira weissflogii*	0.10	0.44	—	
Calanoid copepods assemblage		Heterotrophic ciliate *Uronema* sp.	0.32±0.02	—	—	Twining and Fisher 2004
Calanoid copepods assemblage		Heterotrophic dinoflagellate *Oxyrrhis marina*	0.66±0.06	—	—	
Copepod *Acartia tonsa*		*Thalassiosira oceanica* + Fe‐poor	0.53	0.89	—	Chen et al. 2014
		*Rhodomonas salina* + Fe‐poor	0.43	0.82	—	
		*Isochrysis galbana* + Fe‐poor	0.69	0.87	—	
		*Thalassiosira oceanica* + Fe‐rich	0.47	0.90	—	
		*Rhodomonas salina* + Fe‐rich	0.45	0.87	—	
		*Isochrysis galbana* + Fe‐rich	0.50	0.84	—	
Mixed copepods assemblage		Diatoms + microzooplankton	<0.30	—	0.13–0.25	Sarthou et al. 2008
Brine shrimp *Artemia* sp.		Diatom	—	—	*0.19*	Hutchins and Bruland 1994
Copepod (unidentified)		Diatom	—	—	*0.20*	
Copepod *Acartia tonsa*		Diatom	—	—	*0.07*	
Copepod *Acartia tonsa*		Flagellate	—	—	*0.18*	
Copepod *Acartia tonsa* + cladoceran *Evadne* sp.		Diatom	—	—	*0.22*	
		Flagellate	—	—	*0.10*	
Calanoid copepod *Acartia tonsa*		Diatom *Thalassiosira pseudonana*		—	0.25±0.03	Hutchins et al. 1995
		Diatom *Thalassiosira pseudonana*		—	0.22±0.05	
Calanoid copepods assemblage		Diatoms *Thalassiosira pseudonana*	—	*0.84*	—	Reinfelder and Fisher 1991
Copepod *Calanus pacificus* (adult female)		Diatom *Thalassiosira weissflogii*	—	*0.78±0.07 (0.69–0.85)*	—	Landry et al. 1984
Chaetognath *Sagitta hispida*		Copepods		*0.80 (0.54–0.97)*		Cosper and Reeve 1975
Atlantic salmon, *Salmo salar*		Fish meal or fish meal + wheat gluten diets	*0.11–0.14*	—	—	Storebakken et al. 2000
Atlantic salmon, *Salmo salar*		Feed with fish meal + blood meal + wheat + fish oil	*0.11–0.33 (0.24±0.04)*	—	—	Thodesen et al. 2001
Marine medaka *Oryzias melastigma*	Larval fish	Diets with different Fe supplements	—	—	0.016–0.185	Wang and Wang 2016
	Adult female		—	—	0.009–0.062	
Common sole		Commercial pellets with different Fe sources (haem/non haem Fe)	*0.14–0.17*	—	—	Kals et al. 2016
Damselfish *Chromis chromis*		Plankton (mostly small copepods)		0.84		Pinnegar et al. 2007
Larval *Leiostomus xanthurus* Lacépède		Rotifer *Brachionus plicatilis* + wild plankton		*0.67–0.99*		Govoni et al. 1982

Some of the measurements of absorption and assimilation efficiencies listed in Table 1 may be biased by sloppy feeding, as mentioned above. The experimental values for which sloppy feeding may have altered the determination of absorption or assimilation efficiencies are indicated in italic in Table 1. However, one can note that the assimilation efficiency may also represents the overall net result of all the processes that start from untouched food and produce feces and new matter, including sloppy feeding and absorption across the gut.

### Measured Fe:C in fecal matter vs. food

To test the general expectation that Fe is enriched in fecal matter, we gathered data on the Fe:C in fecal matter and Fe:C in food. We divide the data into three categories (paired, stomach‐paired, and unpaired) depending on the availability of measurements of the Fe:C of food associated with a given fecal matter Fe:C. We define the preferred “paired” category as feeding experiments and experiments where the food was collected at the same time and location as the fecal matter. For the “stomach‐paired” measurements, we use the stomach content Fe:C as an approximation of the Fe:C in the food ingested (Geesey et al. 1984). The “unpaired” measurements, which are the least reliable, use the identified prey mean Fe:C, when referred to in the same study, or the stomach content Fe:C from other studies with the same animal (details and references in Supporting Information Table S1).

**Fig 2:**
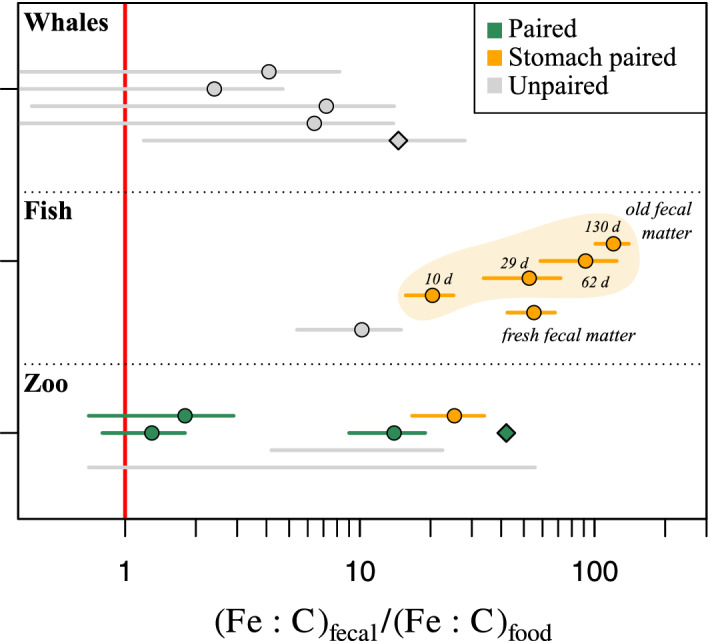
Feces enrichment factor for zooplankton (zoo), fish, and whales. Values are categorized between paired measurements (green points), that is, when in the study Fe content of feces and the food ingested were both measured, stomach‐paired measurements (orange points), that is, when food Fe was not directly measured but undigested stomach content was used instead and, unpaired measurements (gray points), that is, when the food Fe content measurement is not directly related to the food ingested and may come from one or several other studies. Values given without the measurement uncertainty of the feces Fe:C and/or the food Fe:C are plotted as diamonds. The horizontal gray lines with no points are based on a range of values for either Fe:C in feces or food or for both. The vertical red line represents equal Fe:C in feces and food, i.e., no enrichment.

In addition to organic matter, marine animals and their prey can ingest lithogenic particles from which iron can be extracted and absorbed (e.g., Maranger et al. 1998; Schmidt et al. 2011, 2016). Thus, when using ranges of Fe:C in identified prey without including the ingestion of lithogenic or detrital particles, we may be overestimating some of the enrichment factors. Additionally, uncertainty on the Fe:C of the food that is actually ingested may also arise from the release of Fe and C via sloppy feeding as discussed earlier. All the ratios of fecal matter to food Fe:C are represented on Fig. 2 and listed in Supporting Information Table S1.

### Calculation of the Fe:C enrichment of sinking particles due to fecal pellets

The Fe:C enrichment of the particulate organic pool, *E*_part_, that occurs for a given fraction of fecal pellets in the sinking flux, *x*, depends on the Fe:C enrichment of the fecal pellets compared to the Fe:C in food, *E*_FP_ as: *E*_part_ = *xE*_FP_ + (1 − *x*), with *x* the fraction of particulate organic carbon (POC) that is fecal pellets.

**Fig 3:**
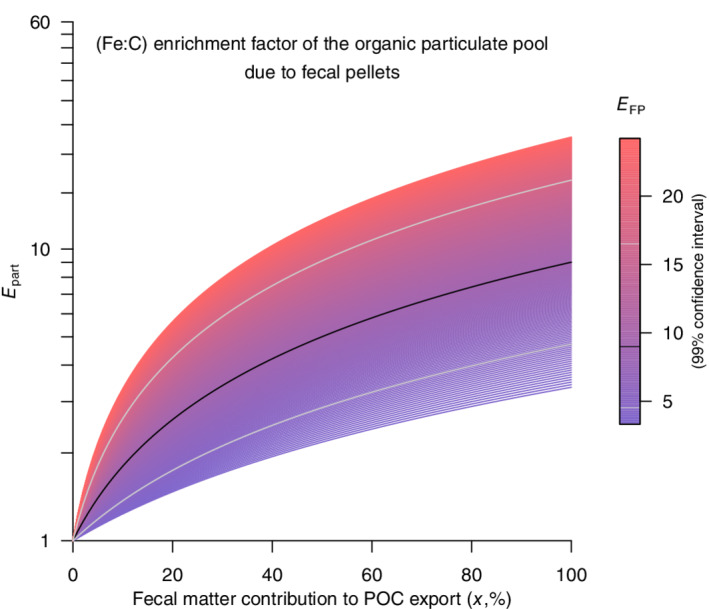
Theoretical estimate of the total Fe:C enrichment factor of the organic fraction of particles as a function of fecal matter contribution to the POC export (x‐axis) and as a function of the fecal matter Fe:C enrichment factor (color). The variable Fe:C enrichment factor of the fecal matter compared to food is based on data from Fig. 2 (excluding old fecal matter) and varies around its mean value (black line on the color bar) at the 99% confidence interval. The colored area on the plot shows the total particulate organic pool enrichment using the variable enrichment factor of fecal matter. The black and gray lines on the plot show the total particulate organic pool enrichment factor trajectory using the mean and 95% confidence interval of the fecal matter enrichment factor, respectively.

The contribution of fecal pellets Fe to the total Fe in the particulate pool is thus:

$\frac{Fe_{\mathrm{FP}}}{Fe_{\text{part}}}=\frac{{\left(\mathrm{Fe}:C\right)}_{\mathrm{FP}}.C_{\mathrm{FP}}}{{\left(\mathrm{Fe}:C\right)}_{\text{part}}.\;\mathrm{POC}}=\frac{{\left(\mathrm{Fe}:C\right)}_{\mathrm{FP}}}{{\left(\mathrm{Fe}:C\right)}_{\text{food}}}\cdot \frac{{\left(\mathrm{Fe}:C\right)}_{\text{food}}}{{\left(\mathrm{Fe}:C\right)}_{\text{part}}}\cdot x=\frac{E_{\mathrm{FP}}}{E_{\text{part}}}\cdot x$, with C_FP_ the fecal pellet carbon content; (Fe:C)_part_, (Fe:C)_FP_, and (Fe:C)_food_ the Fe:C in particles, fecal pellets, and food, respectively. This simple computation is made assuming the Fe:C in nonfecal organic particles is equal to the Fe:C of the animal's food, which ignores the fact that the nonfecal organic particles are composed of more diverse material and subject to degradation by free‐living bacteria. These estimates, shown in Fig. 3, should be seen only as a rough illustration, rather than a precise quantification of the effect on the particulate pool Fe:C.

### Calculation of the Fe nutrition of coprophages

**Fig 4:**
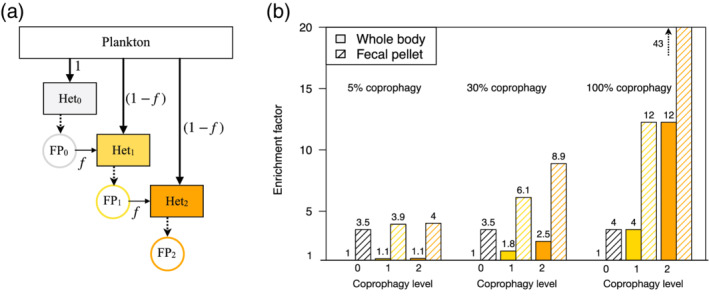
Simple model of how the Fe:C of heterotrophic consumers body (Het) and fecal pellets (FP) could be enriched by coprophagy (coprophagous animals both in orange). Panel (**a**) shows the feeding behaviors of the different heterotrophs (Het). The organism can be of three levels of coprophagy: 0 = not eating any fecal matter (Het_0_), 1 = eating fecal matter from a 0‐level coprophagy organism plus plankton (Het_1_), 2 = eating fecal matter from a level 1 coprophagy organism plus plankton (Het_2_). The fraction of fecal matter included in the diet, *f*, is constant for all coprophages, and is shown for fractions of 5%, 30%, and 100%. The assimilation efficiencies of C and Fe are taken equal to 0.2 and the absorption efficiencies are 0.3 and 0.8 for Fe and C, respectively. The Fe:C in the plankton compartment is fixed. Panel (**b**) shows the enrichment factors of the Fe:C of the body (filled bars) and the fecal pellets (dashed bars) of the different organisms, under the three scenarios and for the different coprophagy levels.

To illustrate how the Fe:C enrichment in fecal matter could supplement the Fe nutrition of coprophagous heterotrophs, we calculate the consequences of variable Fe and C stoichiometry in a simple coprophagous food web (Fig. 4a). We assume that organisms have constant absorption and assimilation efficiencies for Fe of 0.3 and 0.2, respectively, and for C of 0.8 and 0.2, respectively (estimated from Table 1). We define three degrees of coprophagy depending on the dietary fraction of fecal matter and on the feeding behavior of the organism that produced the fecal matter. A coprophagy level of 0 indicates a purely planktivorous diet, a coprophagy level of 1 indicates feeding on a mixture of plankton and fecal matter produced by an organism only eating primary producers, and a coprophagy level of 2 indicates feeding on a mixture of plankton and fecal matter produced by an organism of coprophagy level 1 (Fig. 4a). Thus, the Fe:C of an organism of a coprophagy level *i* is given by:(Fe:C)_body,__*i*_ = copro_*i*_ * (Fe:C)_FP,*i*−1_ + (1 − copro_*i*_) * (Fe:C)_plankton_, where copro_*i*_ is the percentage of the diet composed of fecal pellets, (Fe:C)_FP,__*i*−1_ is the Fe:C of the fecal pellets produced by the organisms of coprophagy level *i* − 1, and (Fe:C)_plankton_ is the Fe:C of plankton.

These feeding behaviors, simplified here, have been observed for instance in coral reef fishes at Palau in the Western Pacific (Bailey and Robertson 1982). We show several scenarios in which we vary the fraction of coprophagy to span the range of observed coprophagic behaviors: 5%, 30%, and 100% of the diet (Fig. 4a) (Frankenberg and Smith 1967; González and Smetacek 1994; Iversen and Poulsen 2007).

## Results and discussion

### Observed enrichment of Fe in feces

#### The contrasting fates of Fe and C in animal guts

Table 1 shows published data for different organisms on the absorption efficiencies of Fe and C, as well as trophic efficiencies of Fe. In general, the absorption of carbon is high, while the absorption of Fe is low (*A*_Fe_ < *A*_C_). In contrast, the trophic efficiency of C is similar to that of Fe (*τ*_C_ ≈ *τ*_Fe_). Thus, while the absorption efficiency of C is high, resulting in most of the absorbed C being excreted as dissolved compounds (*τ*_C_ < *A*_C_, Table 1), the trophic efficiency of Fe is similar to the Fe absorption efficiency (*τ*_Fe_ ≈ *A*_Fe_, Table 1), implying that most of the absorbed Fe is assimilated. Essentially, this basic contrast between the elements reflects the fact that most of the ingested C is respired by the animals to support metabolic needs while there is little difference between the amount of Fe absorbed and the amount retained within the body, that is, only a small fraction of absorbed Fe is excreted back to water as dissolved Fe (Fig. 1; Table 1).

The assimilation data therefore show that, as illustrated in Fig. 1, most of the Fe ingested should transit through the gut and be repackaged into fecal matter while most of the C is absorbed across the gut wall, respired, and excreted to the dissolved phase. The overall expectation is thus for a significant enrichment of the Fe:C to occur in fecal matter compared to the food ingested.

Note that in the framework we described in the “Methods” section, there is no effect of sloppy feeding since we only consider the food that enter the digestive tract. However, since some of the measurements in Table 1 might be biased by sloppy feeding, it would affect the description of the processes such that C is not solely lost via excretion but also during ingestion by sloppy feeding along with some losses of Fe, which does not change our expectations for enriched fecal matter.

#### Measured Fe:C in fecal matter vs. food

Our compilation of measured feces/food pairs confirms the expected enrichment fecal matter Fe:C compared to the ingested food, with all means of the enrichment factors greater than one (Fig. 2). The enrichment of fecal matter Fe:C in paired or stomach‐paired measurements is as low as a factor of 1.3 for copepods fed cultured diatoms (Schmidt et al. 1999) and up to 55 for the fresh feces of blacksmith fish (Geesey et al. 1984). Old fecal matter of fish exhibits the highest enrichment values up to 120 for the oldest fecal material (130‐days old). When averaged across all pairs (excluding old fecal matter and range‐only values), Fe:C in fecal matter is larger than Fe:C in the corresponding food by an order of magnitude (geometric mean of 9.0 ± 3.3). An order of magnitude enrichment would be consistent with an average Fe absorption of 10% and C absorption of 90%. Notably, even though the organisms included in Fig. 1 have different digestive systems, exploit various food sources, and handle their food in different ways (e.g., filterers, mechanical crushing of the prey), their fecal matter appears to be universally enriched in Fe:C compared to their food (Fig. 2).The variability in observed ratios may be explained by a combination of factors, including (1) uncertainty in the Fe:C of the corresponding food source, which can be linked to sloppy feeding as discussed earlier, (2) differences in the elemental absorption efficiencies between animals, and (3) post‐egestion changes in the Fe and C contents as highlighted by changes observed in the aging of fish fecal matter. The next section discusses the latter two factors in greater depth.

### Potential sources of variability in fecal Fe:C

#### Variability in Fe absorption

Marine animals can vary their uptake of Fe to some degree in order to avoid excess Fe. At high concentrations, Fe is toxic and can favor pathogen growth and impair development (e.g., Grosell et al. 2011). While basal losses of Fe occur, no regulated excretory process has been clearly identified (Andersen et al. 1997; Bury and Grosell 2003*a*). It would therefore appear that animals, including humans, can regulate their Fe uptake to avoid toxicity (Papanikolaou and Pantopoulos 2005), but that Fe absorption is limited to a relatively low fraction of the total due to challenges related to Fe chemistry. We do not have a complete understanding of the reasons why the absorption of Fe tends to be low, but we can identify a number of relevant factors affecting absorption that are likely to vary between organisms.

While all organisms use enzymes during digestion, either produced by the animals or by its gut microbiome, to break down complexes and extract essential elements from the food (Donachie and Zdanowski 1998; Grosell et al. 2011; Freese et al. 2012), some animals also possess gastric glands and can operate an acid lysis of the food cells to release their content (Grosell et al. 2011; Štrus et al. 2019). In addition, upon ingestion some animals are able to mechanically release the Fe from their preys by crushing them in their foregut and/or triturating the food in their gut (Grosell et al. 2011; Štrus et al. 2019).

Once Fe is released from the food, it must be prevented from precipitating and absorbed across the gut epithelium, which can be difficult depending on the gut environment. For example, the osmotic regulation of fish produces bicarbonate that binds to Fe and precipitates in the form of iron carbonates, thus increasing the difficulty of extracting iron from the food (Bury and Grosell 2003*a*; Grosell et al. 2011). Other elements present in the food such as copper may interfere with the absorption of Fe as it uses the same route of absorption (Kals et al. 2016), while others such as ascorbic acid may enhance Fe transfer to the gut epithelium (Cooper et al. 2006). A low pH in the gut of animals with gastric glands reduces the oxidation rate of Fe^2+^ (Liu and Millero 2002), and more importantly the presence of low oxygen zones in the gut (e.g., for copepods, Tang et al. 2011) forces the reduction of Fe^3+^ to Fe^2+^, the latter of which is more bioavailable and binds to specific proteins in order to cross the gut epithelium of fish for example (Cooper et al. 2006). In addition, secretions such as mucus in fish gut that binds to Fe^2+^ and Fe^3+^, and the presence of ligands, either ingested or produced by the gut microbiome, help maintain Fe in solution (Tortell et al. 1999; Cooper et al. 2006; Hunter and Boyd 2007). Inversely, ingested pathogens and nonresident bacteria present in the gut (e.g., Tang et al. 2010) may compete for Fe, thus reducing its absorption.

In addition to variations caused by organism‐specific digestive features, experiments have shown that Fe absorption depends on the form and distribution of Fe within the food. For example, the Fe absorption efficiency of copepods increases linearly with the Fe content of the cytoplasm of their prey, while Fe bound to the exoskeleton or cell membrane appears more difficult to absorb (Chen et al. 2014). Heme Fe, that is, bound within Fe‐porphyrin complexes found in widespread metalloproteins including hemoglobin and myoglobin (Hogle et al. 2014), is more efficiently absorbed than nonheme Fe by fish (Andersen et al. 1997). In some cases, the absorption efficiency of Fe has also been shown to vary with the Fe concentration in food, with a higher absorption when the food had a lower Fe content (Andersen et al. 1997; Schmidt et al. 1999).

As a result of the multiplicity of parameters affecting Fe absorption, different taxa may tend to have different absorption efficiencies, as suggested for example by the more efficient Fe absorption by zooplankton than by fish shown in Table 1. In addition, Wang and Wang (2016) showed that the Fe absorption of the marine medaka *Oryzias melastigma* was significantly different between larval and female adult fish, thus highlighting a possible effect of age on the regulation of Fe absorption, which would also be coherent with the fact that larvae are building their Fe stores and need to absorb more Fe to meet their requirements compared to adults (Galbraith et al. 2019).

In summary, the low Fe absorption of marine animals relative to their C absorption, which is paramount in driving the Fe:C enrichment of feces, arises from a combination of factors ranging from Fe chemical speciation to organism‐specific ingestion and digestion.

#### Post‐egestion modification of fecal matter Fe:C

After egestion, the Fe:C of fecal matter is likely to vary as it exchanges with its environment, so that the measured Fe:C of fecal matter could differ from the Fe:C it would have had when originally expelled. Apart from the inherent fragility or resistance of fecal matter (e.g., partly liquid whale feces; Roman et al. 2014), physical and biological mechanical degradation of the fecal matter may occur and accelerate its recycling, thus modifying its Fe:C.

The release of both Fe and C from fecal matter is slower when the physical integrity of the fecal particles, that is, the fecal pellets, is maintained. The presence of a peritrophic membrane that maintains the integrity of the fecal pellets and favors their export to depth is likely limiting exchanges with the environment (Frangoulis et al. 2004), when it is not degraded (Lautenschlager et al. 1978) or eaten by organisms (Lampitt et al. 1990). Without any perturbations, Hutchins et al. (1995) showed that the copepod fecal pellets retained most of their Fe (> 80%) over 30 days and similarly, Cabanes et al. (2017) showed that Fe in salp fecal pellets was highly refractory. If they are instead disrupted by animals, for example, via coprohexy or coprochaly, fecal particles release Fe and organic C to the dissolved pool (Turner 2015), and the fragmentation of fecal particles into smaller pieces allows for a more efficient recycling of Fe by bacteria returning Fe more rapidly to the water column (e.g., Noji et al. 1991; Iversen and Poulsen 2007).

More important for the stoichiometry of sinking particles are interelement differences in the ways in which Fe and C exchange with the environment. The release of organic C from fecal pellets has been shown to be fast due to the presence of soluble carbon compounds (Jumars et al. 1989) and more importantly, it is faster than the release of Fe in fecal pellets and sinking particles (Hutchins et al. 1995; Frew et al. 2006; Twining et al. 2014), which should contribute to increasing Fe:C in fecal pellets over time and with depth. In addition, fecal pellets may scavenge dissolved Fe from the water column as they sink to depth, which increases their Fe:C with time, as evidenced by Geesey et al. (1984), whose work on fish fecal pellets showed that old feces contained more Fe than fresh ones (Fig. 2). At the same time, this scavenging may be counteracted to some degree by the release of ligands from the fecal pellets either by the bacteria attached to it or via the disruption of the pellet (Cabanes et al. 2017; Laglera et al. 2020), which would keep Fe in solution close to the pellet and limit its adsorption. Quantifying the relative importance of these factors will require future observational studies.

### The contribution of fecal pellets to the Fe:C of export

Fecal pellets have been shown to be an important component of the carbon flux in the ocean mostly due to their large sinking speed, which tends to be positively related to their size and density (Wotton and Malmqvist 2001). The contribution of zooplankton fecal pellets to the sinking POC flux depends on the time of year, location, rate of pellet degradation, productivity, and community composition, and can represent as little as < 1% or as much as 100% of the sinking flux of POC (e.g., Turner 2015; Steinberg and Landry 2017; Belcher et al. 2019). Fish fecal pellets can also be an important vector of POC in the water column. For example, up to 17% of POC captured in sediment traps in the Peruvian coastal upwelling was from anchoveta fecal pellets (Staresinic et al. 1983), while 15–17% of the POC export is estimated to be mediated by mesopelagic fishes (including active transport) off of Southern California and up to 40% in the North Pacific Subtropical Gyre (Davison et al. 2013). Given that fecal pellets are a globally significant vector for the downward transport of carbon, their high Fe:C implies that they could dominate the downward transport mechanism for organic Fe in many parts of the ocean.

A rough estimate for how much the presence of Fe‐rich fecal pellets could enrich the Fe:C of the organic fraction of sinking particles is shown in Fig. 3. For example, if fecal pellets contribute to 30% of the sinking flux of POC, the Fe:C enrichment of the particulate organic pool (*E*_part_) would range between 1.4‐ and 8‐fold based on the 99% confidence interval around the geometric mean of the observed Fe:C enrichment of the fecal pellets (*E*_FP_, color‐scale on Fig. 3); using the observed geometric mean of *E*_FP_ (*E*_FP_ = 9.0), the mean enrichment of organic particles by the fecal Fe pump would be 3.4‐fold (Fig. 3). Under this scenario (i.e., a 30% contribution of fecal pellets to the sinking flux of POC), between 50% and 91% of the organic sinking flux of Fe would be due to fecal pellets (with a mean value of 80% using the mean observed *E*_FP_).

#### Sources of variability in the fecal Fe pump importance

The contribution of fecal pellets to the biogenic fraction of particulate Fe is bound to be proportionally larger than the overall effect on sinking fluxes, due to the presence of lithogenic and authigenic Fe fractions. Unfortunately, measuring the biogenic Fe in sinking particles is challenging and the results are methodologically dependent (Rauschenberg and Twining 2015). Many studies assume that the Fe:P or Fe:C ratio is constant in biological material, and use the Fe:P or Fe:C of phytoplankton to compute the Fe biogenic profiles (e.g., Bowie et al. 2015; Barrett et al. 2018). Obviously, assuming a constant Fe:P or Fe:C ratio will prevent the accurate determination of changes in Fe stoichiometry such as those identified here. Observed profiles of biogenic particulate Fe worldwide are highly variable, depending on the station and on the time of the measurements (e.g., Planquette et al. 2011), adding another difficulty to testing the contribution of marine animals to the Fe:C variations of sinking particulate matter.

Complicating the matter further, krill have been shown to ingest lithogenic particles and to extract Fe from them (Schmidt et al. 2016), and flagellates can incorporate lithogenic Fe via phagotrophy (Maranger et al. 1998), which is then transferred to marine animals upon grazing. This suggests that animals are also likely to contribute to the redistribution of particulate lithogenic Fe. Given these complications, it is difficult to provide a confident estimate for how the fecal Fe pump compares to the total non‐fecal Fe flux.

Vertical migrations and egestion at depth likely affect the importance of fecal pellets in shaping the particulate Fe:C distribution in the water column as these processes can significantly contribute to the POC export (e.g., Boyd et al. 2019). In the Southern Ocean, the flux of fecal pellets at depth has been shown to increase due to in situ production of fecal pellets in the mesopelagic and bathypelagic layers (Belcher et al. 2017), which would increase the particulate enrichment factor with depth. Reverse fluxes from the seabed to shallow layers by organisms feeding on the sediment may also add some variability in the contribution of fecal matter to the Fe enrichment of particles (Schmidt et al. 2011).

Community composition is an important factor in determining the contribution of fecal pellets to the sinking fluxes of organic carbon as it influences the remineralization length scale of the particles. Indeed, the fecal pellets can be mostly consumed in the epipelagic, as evidenced for example by the presence of copepods that highly increases the recycling of fecal material in the upper layers of the ocean (e.g., Sarthou et al. 2008; Laglera et al. 2017). In addition to the ingestion of fecal pellets, that is, coprophagy (González and Smetacek 1994), the fragmentation of fecal pellets (Iversen and Poulsen 2007) also reduces their contribution to the sinking flux of particles, directly or through faster bacterial remineralization due to reduced sinking speeds. Finally, the reprocessing of sinking particles at depth through coprophagy also produces new fecal pellets, themselves more enriched in Fe compared to C.

### Coprophagy and Fe‐rich fecal matter

The results in Fig. 4b show the degree to which the Fe:C enrichment in the body and fecal matter of an organism would be expected to increase with the percentage of coprophagy and with the coprophagy level, for the illustrative absorption and assimilation rates. The Fe:C increase is greater with a higher consumption of fecal matter in the diet, from 1.1‐fold (1.1‐fold) body‐enrichment with 5% coprophagy to 3.5‐fold (12‐fold) body‐enrichment with 100% coprophagy for a coprophagy level 1 (level 2) animal.

Reality is sure to be much more nuanced that this simple theoretical calculation indicates, yet the implied Fe:C enrichment of fecal matter is quite conservative compared to the fecal matter Fe:C enrichment values computed from published data (Fig. 2), which shows a mean enrichment of about one order of magnitude. These numerical results illustrate the large degree to which the consumption of fecal matter could provide Fe nutrition to marine animals.

In addition to coprophagy, the ingestion of smaller organisms that feed on or incorporate the enriched fecal pellets and sinking particles via phagotrophy (Maranger et al. 1998; Twining and Fisher 2004) may also be an important pathway through which this Fe‐rich source food is transferred to marine animals.

#### Fe bioavailability in fecal matter

Our data compilations and calculations both show that Fe:C can be strongly enriched in fecal matter, but the degree to which the fecal Fe pump may provide an important source of nutrition depends on the bioavailability of fecal Fe. Some aspects of fecal matter formation would be expected to contribute to low Fe bioavailability, while others should raise the bioavailability. On one hand, easily absorbed Fe could be expected to be assimilated first, leaving more refractory Fe to be routed to the fecal matter (Hutchins et al. 1995). On the other hand, Fe in fecal matter can be accessible to other organisms for absorption. For instance, Schmidt et al. (2016) showed that the proportions of labile iron in krill fecal pellets was five times higher than in diatoms, and Sarthou et al. (2008) and Laglera et al. (2017) concluded that the recycling of Fe from copepod fecal pellets is responsible for longer phytoplankton blooms, thus suggesting that Fe in the fecal matter is bioavailable. Fe bioavailability in fecal matter is affected by multiple factors such as the presence of undigested cells and organisms (e.g., Aarnio and Bonsdorff 1997; Friedland et al. 2005; Köster et al. 2011), the presence of an acidic phase in the digestion process that can modify Fe speciation and help extract Fe from lithogenic particles (e.g., Schmidt et al. 2016), the presence of ligands that keep Fe in solution and potentially available (e.g., Nuester et al. 2014; Laglera et al. 2020). In addition, the microbial community within the pellet matrix and attached to its surface grows and degrades the fecal matter during its sojourn in the water column (e.g., Tang et al. 2010; Morata and Seuthe 2014) and may alter the Fe state within the feces through the release of ligands and low oxygen concentrations within the fecal matter, as in aggregates (Balzano et al. 2009). Microbes that grow on the fecal matter, attached to the peritrophic membrane, may be digested when the fecal matter is ingested (Newell 1965; Lampitt et al. 1990; Anderson et al. 2017), potentially constituting an additional source of bioavailable Fe.

In short, although work is needed to have a clearer understanding of the Fe bioavailability of fecal matter to different marine animals, it would appear likely that it is generally a rich source of bioavailable Fe.

## Conclusions

In this article, we have shown that the fecal matter of marine animals is generally enriched in Fe:C compared to their food, primarily because animals digest and respire most of the carbon they ingest, while allowing most of the Fe to pass through and be packaged as fecal matter. This low absorption efficiency of Fe appears to depend on multiple factors related to the physiology of the organism and the type of food it consumes. The digestive enrichment can then be supplemented through the additional processes that preferentially allow organic C to leak from the fecal pellets, as well as the potential scavenging of Fe by the fecal pellets. The sinking of this fecal matter therefore enriches the Fe:C of particles at depth, a process we term the fecal Fe pump. Given the significant contribution of sinking fecal matter to the export of organic carbon, combined with its high observed Fe content, we hypothesize that the fecal Fe pump is a dominant pathway by which biogenic Fe is preferentially exported to depth, relative to other nutrients, at many places in the ocean. This hypothesis could be further tested with new measurements that are able to distinguish changes in the Fe:C of organic matter within the water column.

Moreover, we propose that the fecal matter serves as a Fe‐rich food source to marine animals via coprophagy, especially relevant for the mesopelagic community. This could contribute to the relatively high abundance of mesopelagic fish in high nutrient, low chlorophyll regions where the pelagic biomass of fish seems to be limited by the Fe content of their prey.

The apparent importance of the fecal Fe pump raises many questions about how it may respond to anthropogenic change. In the context of climate change, rising ocean temperature may accelerate fecal matter degradation and/or shift the community to smaller animals producing smaller particles (Heneghan et al. 2019), thereby decreasing the fecal Fe pump effectiveness. On the other hand, community changes such as a shift from krill to salps in the Southern Ocean are predicted (Atkinson et al. 2004), which might increase Fe transport to depth via heavier, poorly digested fecal matter (Cabanes et al. 2017). At the same time, declining oxygen concentrations that affect the respiration rates of organisms, and fishing activity that removes large organisms and modifies ecosystem structures and communities (Getzlaff and Oschlies 2017), might also impact the fecal Fe pump in ways that are challenging to predict. Resolving these questions will require new observations from the field as well as experimental and modeling studies.

## Acknowledgements

We thank the editors and both reviewers for their helpful comments. This study was supported by the European Research Council (ERC) under the European Union's Horizon 2020 Research and Innovation Program (Grant Agreement No 682602) and the Spanish Ministry of Science, Innovation and Universities, through the “María de Maeztu” Program for Units of Excellence (MDM‐2015‐0552).

## Conflict of Interest

None declared.

## Supporting information


**Supplementary Table S1** Collection of measurements of Fe content of feces and food for different organisms and their classification as “paired”, “stomach paired” or “unpaired” measurements.
