# Supplementary material for: The fecal iron pump: Global impact of animals on the iron stoichiometry of marine sinking particles
Source: Limnol Oceanogr. 2020 Oct 6;66(1):201–13. doi: 10.1002/lno.11597 (PMC7891356; doi:10.1002/lno.11597)
Supplement: Supplementary file 1 — Supplementary Table S1 Collection of measurements of Fe content of feces and food for different organisms and their classification as “paired”, “stomach paired” or “unpaired” measurements. [file LNO-66-201-s001.pdf]

Supplementary Information for **"The fecal iron pump: global impact of animals on the iron stoichiometry of marine sinking particles"**

Supplementary Table S1: Collection of measurements of Fe content of feces and food for different organisms and their classification as "paired", "stomach paired" or "unpaired" measurements.

Location	Time	Depth	Conditions		Organism common name	Organism latin name	Feces Fe content	Original feces units	Food item	Food Fe content	Original food units	Classification	Comment	FP/food	References for feces	References for food
Kelp beds off Pacific Grove, California			Incubations 3-7 days		Crab	*Pugettia producta*	1021 +/- 367	µgFe/g ash	Brown algae (*Macrocystis pyrifera*)	73 +/- 5	µgFe/ash	paired		14 +/- 5	Boothe and Knauer, 1972	
Surface waters off Monaco		surface	15h depuration		euphausiids	*Meganyctiphanes norvegica*	24000	µgFe/g dry matter	Microplankton (copepods, phytoplankton, chaetognaths and detritus)	570	µgFe/g dry matter	paired	no uncertainty on feces and food measurements	42.1	Fowler, 1977	
Rocky bottom off Santa Catalina Island (USA)	July 1981	Rocky bottom	In-situ collection buckets and nocturnal shelters incubation	fresh	Blacksmith	*Chromis punctipinnis*	7170 +/- 1316	µgFe/g dry matter	Undigested food from the stomach	130 +/- 18	µgFe/g dry matter	stomach paired		55.2 +/- 12.7	Geesey et al., 1984	
				10 days			2652 +/- 496							20.4 +/- 4.7		
				29 days			6856 +/- 2285							52.7 +/- 19.0		
				62 days			11914 +/-3940							91.6 +/- 32.8		
				130 days			15650 +/- 1344							120.4 +/- 19.6		
Head of Salt River Submarine Canyon off St. Croix, US Virgin Islands		15m		fresh	Brown chromis	*Chromis multilineatus*	1320 +/- 594	µgFe/g dry matter	Undigested food from the stomach of blackmith	130 +/- 18	µgFe/g dry matter	unpaired		10.2 +/- 4.8		
Delaware Bay	-	-	Cultured Fe limited food		calanoid copepods	*Arcartia spp.*	30 +/- 15	µmolFe/molC	*T. weissflogii*	17 +/- 6	µmolFe/molC	paired	Food intracellular Fe only	1.8 +/- 1.1	Schmidt et al., 1999	
Delaware Bay	-	-	Cultured Fe replete food		calanoid copepods	*Arcartia spp.*	46 +/- 15	µmolFe/molC	*T. weissflogii*	35 +/- 6	µmolFe/molC	paired		1.3 +/- 0.5		
Southern Ocean				n=1	Sperm whale	*Physeter macrocephalus*	467.4	µmolFe/molC	4 krill species mean Fe:C	32.1 +/- 29.5	µmolFe/molC	unpaired	no uncertainty on feces measurement	14.6 +/- 13.4	Ratnarajah et al., 2014	
				n=15	Blue whale	*Balaenoptera musculus*	206.8 +/- 148.5					unpaired		6.4 +/- 7.5		
				n=2	Fin whale	*Balaenoptera physalus*	230.2 +/- 48.2					unpaired		7.2 +/- 6.8		
				n=2	Humpback whale	*Megaptera novaeangliae*	132.7 +/- 45.8					unpaired		4.1 +/- 4.1		
				n=7	Pygmy blue whale	*Balaenoptera musculus brevicauda*	76.5 +/- 14.3					unpaired		2.4 +/- 2.3		
					Mean without Humpback whale		182.7 +/- 142.2					unpaired		5.7 +/- 6.9		
Subantarctic Snares and Auckland Islands					Southern right whale		~ 370	µmolFe/molC	Large zooplankton, krill	32.1 +/- 29.5	µmolFe/molC	unpaired	no uncertainty on feces measurement	11.5 +/- 10.6	Wing et al., 2014	Ratnarajah et al., 2014
Pryzd Bay, Antarctica					Antarctic krill	*Euphausia superba*	861 +/- 252	mgFe/kg dry matter	Stomach content	34 +/- 6	mgFe/kg dry matter	stomach paired		25.3 +/- 8.6	Ratnarajah et al., 2016	
South Gerogia in the Southern Ocean	dec 2010-jan 2011		Shipboard incubations		Antarctic krill	*Euphausia superba*	2-149	mgFe/gdry matter	Stomach content (labile)	111.2	µgFe/g dry matter	unpaired	**Fecal pellet Fe content is the total particulate Fe so with refractory Fe**	17.8-1330	Schmidt et al., 2016	Schmidt et al., 2011)
									Stomach content (refractory)	2672		unpaired		0.7-55.8		
Atlantic sector of the Southern Ocean	Jan-March 2012	Salps (25m) - Fecal pellets flux (100m, 300m)	Incubation tanks, leaching 2 months at pH 1.5		Salps	*Salpa thompsoni*	0.33 (225)	nmolFe/pellet (µmolFe/molC)	Plankton in HNLC waters	10-50	µmolFe/molC	unpaired	ranges	4.2-22.5	Cabanes et al., 2017	Cabanes et al., 2017 from Twining and Baines (2013) and Boyd et al. (2015)

References

Boothe, P. N., and G. A. Knauer. 1972. The possible importance of fecal material in the biological amplification of trace and heavy metals. Limnol. Oceanogr. **17**: 270–274.

Boyd, P. W., R. F. Strzepek, M. J. Ellwood, D. A. Hutchins, S. D. Nodder, B. S. Twining, and S. W. Wilhelm. 2015. Why are biotic iron pools uniform across high- and low-iron pelagic ecosystems? Global Biogeochem. Cycles **29**: 1028–1043. doi:10.1002/2014GB005014

Cabanes, D. J. E., L. Norman, J. Santos-Echeandía, M. H. Iversen, S. Trimborn, L. M. Laglera, and C. S. Hassler. 2017. First Evaluation of the Role of Salp Fecal Pellets on Iron Biogeochemistry. Front. Mar. Sci. **3**. doi:10.3389/fmars.2016.00289

Fowler, S. W. 1977. Trace elements in zooplankton particulate products. Nature **269**: 51–53. doi:10.1038/269051a0

Geesey, G., G. Alexander, R. Bray, and A. Miller. 1984. Fish fecal pellets are a source of minerals for inshore reef communities. Mar. Ecol. Prog. Ser. **15**: 19–25. doi:10.3354/meps015019

Ratnarajah, L., A. R. Bowie, D. Lannuzel, K. M. Meiners, and S. Nicol. 2014. The biogeochemical role of baleen whales and krill in Southern Ocean nutrient cycling. PLoS One **9**: 1–18. doi:10.1371/journal.pone.0114067

Ratnarajah, L., S. Nicol, S. Kawaguchi, A. T. Townsend, D. Lannuzel, K. M. Meiners, and A. R. Bowie. 2016. Understanding the variability in the iron concentration of Antarctic krill. Limnol. Oceanogr. **61**: 1651–1660. doi:10.1002/lno.10322

Schmidt, K., A. Atkinson, S. Steigenberger, and others. 2011. Seabed foraging by antarctic krill: Implications for stock assessment, bentho-pelagic coupling, and the vertical transfer of iron. Limnol. Oceanogr. **56**: 1411–1428. doi:10.4319/lo.2011.56.4.1411

Schmidt, K., C. Schlosser, A. Atkinson, S. Fielding, H. J. Venables, C. M. Waluda, and E. P. Achterberg. 2016. Zooplankton Gut Passage Mobilizes Lithogenic Iron for Ocean Productivity. Curr. Biol. **26**: 2667–2673. doi:10.1016/j.cub.2016.07.058

Schmidt, M. A., Y. Zhang, and D. A. Hutchins. 1999. Assimilation of Fe and carbon by marine copepods from Fe-limited and Fe-replete diatom prey. J. Plankton Res. **21**: 1753–1764. doi:10.1093/plankt/21.9.1753

Twining, B. S., and S. B. Baines. 2013. The Trace Metal Composition of Marine Phytoplankton. Ann. Rev. Mar. Sci. **5**: 191–215. doi:10.1146/annurev-marine-121211-172322

Wing, S. R., L. Jack, O. Shatova, J. J. Leichter, D. Barr, R. D. Frew, and M. Gault-Ringold. 2014. Seabirds and marine mammals redistribute bioavailable iron in the Southern Ocean. Mar. Ecol. Prog. Ser. **510**: 1–13. doi:10.3354/meps10923
